# Elucidating the Interplay of Hypoxia-Inducible Factor and Circadian Clock Signaling in Obstructive Sleep Apnea Patients

**DOI:** 10.3390/ijms26030971

**Published:** 2025-01-24

**Authors:** Agata Gabryelska, Szymon Turkiewicz, Adrian Gajewski, Piotr Białasiewicz, Dominik Strzelecki, Marta Ditmer, Maciej Chałubiński, Marcin Sochal

**Affiliations:** 1 Department of Sleep Medicine and Metabolic Disorder, Medical University of Lodz, 92-215 Łódź, Poland; szymon.turkiewicz@umed.lodz.pl (S.T.); piotr.bialasiewicz@umed.lodz.pl (P.B.); marta.ditmer@umed.lodz.pl (M.D.); marcin.sochal@umed.lodz.pl (M.S.); 2 Department of Immunology and Allergy, Medical University of Lodz, 92-213 Łódź, Poland; adrian.gajewski@umed.lodz.pl (A.G.); maciej.chalubinski@umed.lodz.pl (M.C.); 3 Department of Affective and Psychotic Disorders, Medical University of Lodz, 92-216 Łódź, Poland; dominik.strzelecki@umed.lodz.pl

**Keywords:** circadian clock, OSA, intermittent hypoxia, HIF-1 alpha, HIF-1 beta, BMAL1, CLOCK, PER1, CRY1

## Abstract

Background: Hypoxia-inducible factor 1 (HIF-1) affects the circadian clock in obstructive sleep apnea (OSA) and may have a bidirectional relationship with circadian mechanisms. This study examined the link between circadian clock and HIF-1 in OSA patients versus controls. Methods: 70 participants underwent polysomnography (PSG), and were assigned into OSA (apnea–hypopnea index (AHI) ≥ 5, n = 54) or control (AHI < 5, n = 16) groups. *BMAL1* (brain and muscle ARNT like 1), *CLOCK* (circadian locomotor output cycles kaput), *PER1* (period 1), *CRY1* (cryptochrome 1), *HIF-1α*, and *HIF-1β* gene expressions and protein levels were measured in evening and morning samples, collected before and after PSG. Results: The OSA group was characterized by increased CLOCK, CRY1, PER1 and HIF-1a protein levels, both in the morning and evening (all *p* < 0.05), and decreased morning expression of *BMAL1* (*p* = 0.02). Associations between almost all circadian clock gene expressions and both HIF-1 subunits were observed in the OSA group at both time points (all *p* < 0.05), apart from association between PER1 and HIF-1α in the morning (R = 0.050, *p* = 0.73). In controls, only a correlation between HIF-1α levels and *CRY1* expression in the morning (R = 0.588, *p* = 0.02) was found. Conclusions: OSA affects the circadian clock and HIF-1 pathway, with increased CLOCK, CRY1, PER1, and HIF-1α protein levels observed in OSA patients. The interplay between these systems may involve complex posttranscriptional and posttranslational mechanisms.

## 1. Introduction

Obstructive Sleep Apnea (OSA) is a common condition marked by repeated instances where the upper respiratory tract is either partially or fully blocked during sleep. This obstruction results in diminished airflow or a complete halt, even as the individual continues to attempt to breathe. OSA is the most prevalent form of sleep-disordered breathing, characterized by repeated episodes of apneas and hypopneas during sleep, leading to fragmented sleep and reduced oxygen levels and significantly impacting overall health and quality of life [1]. This condition is associated with intermittent hypoxia (IH) and sleep fragmentation, which can lead to various comorbidities and disruption in the circadian clock [2,3].

A pivotal molecular reaction to the intermittent hypoxia (IH) associated with OSA is mediated by hypoxia-inducible factors (HIFs), comprising two subunits: HIF-1α and HIF-1β. These transcription factors are essential in how cells respond to oxygen availability. HIF-1α and HIF-1β combine to create a heterodimer that attaches to hypoxia-responsive elements (HREs) located in the promoter areas of certain genes. This action triggers the activation of genes critical for processes such as the formation of new blood vessels, metabolism regulation, the production of red blood cells, and mechanisms that support cell survival [4,5].

The circadian clock, an endogenous time-keeping system, regulates physiological processes through two feedback loops. The first one includes activators (BMAL1, brain and muscle ARNT-like 1; CLOCK, circadian locomotor output cycles kaput) and repressors (PER, period; CRYs, cryptochrome). The BMAL1: CLOCK complex acts as a transcription factor and binds to genes with E-box motifs in their promotors, stimulating expression of repressors and other genes, such as *HIF-1α*, *RORα* (RAR-related orphan receptor A) and *NR1D1* (nuclear receptor subfamily 1 group D member 1). The repressors, in turn, bind to each other and show inhibitory effects on activators. The second loop involves the regulation of *BMAL1* expression by RORα and NR1D1, which stimulate and inhibit it, respectively [3,6].

The circadian system and HIF pathways interact at the molecular level, influencing each other’s expression and activity in a bidirectional way [3,6]. Circadian clock repressor genes are the targets of the HIF-1 transcription factor complex, due to the presence of HRE in their promotor. Similarly, circadian clock activators may induce transcription of *HIF-1α*. Moreover, HIF-1α binds to BMAL1, replacing CLOCK [3,7]. Preliminary reports provide data suggesting the presence of disruption of the circadian clock signaling pathway in OSA patients that might be mediated by HIF-1.

Thus, this study aims to explore the relationship between circadian clock gene expressions, their protein products, and HIF-1 subunits in patients with OSA compared to a control group without the condition. By analyzing serum protein levels and gene expressions of key circadian and HIF-1 components, we seek to elucidate the potential impact of OSA-induced IH on the circadian system and its interaction with the HIF-1 pathway.

## 2. Results

The control and OSA groups comprised 16 and 54 participants, respectively, with no significant differences observed in age (*p* = 0.53), BMI (body mass index, *p* = 0.14), or sex (*p* = 0.34) between the groups. The lack of significant disparities in these key variables ensures the validity of comparisons between the two cohorts. All demographic data and polysomnography (PSG) parameters are presented in Table 1.

Out of all circadian clock gene expression levels, only morning *BMAL1* was statistically significantly lower in the OSA compared to the control group (*p* = 0.02, 6.31 vs. 14.53). Furthermore, in both the control and OSA groups, morning *PER1* gene expressions were greater than in the evening (*p* = 0.02, 2.05 vs. 6.90 and *p* = 0.004, 2.63 vs. 7.45, respectively).

OSA patients presented with higher evening and morning CLOCK (*p* = 0.03 and *p* = 0.008, respectively), CRY1 (*p* = 0.048 and *p* = 0.003, respectively), and PER1 (*p* = 0.003 and *p* = 0.02, respectively) protein levels.

Regarding HIF-1 subunits, only the morning HIF-1α protein level was higher in OSA participants compared to the control group.

All comparisons between circadian clock gene and HIF-1 subunit gene expression and protein levels are presented in Table 2.

No statistically significant correlations between gene expression and protein levels were observed either in the morning or in the evening in the case of all evaluated circadian clocks (all *p* > 0.20) among the OSA and control groups.

In the OSA group, statistically significant associations between evening and morning *CLOCK*, *CRY1*, and *PER1* gene expressions were found (R = 0.400, *p* = 0.02; R = 0.468, *p* = 0.007 and R = 0.528, *p* < 0.001, respectively), while for *BMAL1* only a tendency was present (R = 0.296, *p* = 0.06). On the other hand, in the control group, no such relationships were observed (all *p* > 0.45).

In regard to correlations between evening and morning circadian clock protein levels, in the OSA group, they were found for CLOCK (R = 0.600, *p* < 0.001), CRY1 (R = 0.440, *p* < 0.001), and PER1 (R = 0.583, *p* < 0.001)—all are statistically significant—but not for BMAL1 (R = 0.057, *p* = 0.68). In the control group, statistically significant relationships were present in CLOCK (R = 0.636, *p* = 0.008) and PER1 (R = 0.532, *p* = 0.03) but not in BMAL1 (R = 0.414, *p* = 0.11) or CRY1 (R = 0.203, *p* = 0.45).

In the OSA group, on the gene expression level, all analyzed circadian clock genes correlated at a given time point (evening/morning) with HIF-1α and HIF-1β gene expression; the only exception was a lack of association between PER1 and HIF-1α in the morning (R = 0.050, *p* = 0.73). On the other hand, in the control group, no correlations between the circadian clock and HIF-1α gene expression were present in the morning, and only CLOCK and CRY1 were associated with HIF-1α in the evening (R = 0.57, *p* = 0.03, and R = 0.552, *p* = 0.04, respectively). All correlations between circadian clock gene expressions and protein levels in the evening and the morning in the OSA and control groups are presented in Table 3.

## 3. Discussion

Our investigation into the intricate relationship between circadian clock protein levels and HIF-1 in OSA individuals presents novel insights. This research stands as the first to assess circadian clock gene expressions and their protein product levels in OSA at the same time.

As we expected, CLOCK, CRY1, PER1, and HIF-1α protein levels were increased in OSA individuals, which is similar to our result from the preliminary study [8]. Moreover, PER1 protein levels increased in the morning compared to the evening in both groups. To date, there are no other studies investigating the circadian clock protein levels in OSA that may be discussed. However, upregulation of HIF-1α has already been repeatedly demonstrated in numerous studies on mice [9,10,11,12] and humans [13,14,15] in the context of OSA or IH, at both the protein and gene expression levels. HIF-1α can affect the regulation of the circadian clock in several ways. Initially, HIF-1α associates with cofactors such as HIF-1β and p300 to constitute an operative transcriptional complex, targeting genes with HRE in their promoters, including genes encoding PER and CRY proteins. Under intermittent hypoxic conditions, elevated HIF-1α levels may augment the expression of certain repressors. Furthermore, HIF-1α is posited to supplant CLOCK’s role, associating with BMAL1 to facilitate the transcription of genes traditionally activated by circadian regulators [16,17]. This bidirectional mechanism between the circadian clock and HIF-1α may explain the positive correlations between the expression of almost all circadian genes and HIF-1 subunits. However, despite these mechanisms, our investigation revealed no variance in *PER1* and *CRY1* gene expression between groups, notwithstanding significant disparities in protein concentrations.

In the context of OSA, discrepancies have emerged in the research on circadian clock gene expression. Some studies, such as those by Gaspar et al., have identified alterations in the expression of key circadian genes, including increased *BMAL1* and decreased *PER1* and *CRY2*, suggesting a disruption in circadian rhythms among OSA patients [18]. These disruptions vary, with some findings indicating increased expression of certain genes during specific times and reductions in others, alongside the loss of diurnal expression patterns [19,20,21,22]. For example, in research conducted by Yang et al., an elevation in the expression levels of *BMAL1*, *CLOCK*, *CRY1*, and *PER3* was observed during the evening hours. Furthermore, the study demonstrated the elimination of diurnal rhythmicity in the expression of *BMAL1*, *CLOCK*, *CRY2*, and *PER1* genes [19]. Additional investigations have corroborated these findings, indicating a reduction in the expression levels of *CLOCK* [21] and *PER3* [22]. Notably, one particular study elucidated that patients suffering from OSA with nocturnal hypoxemia (NH), defined as a state where ≥10% of the total sleep duration is characterized by an oxygen saturation level below 90%, exhibited diminished expression of *NR1D1* and *PER1* in comparison to those without NH [22]. Moreover, mouse models of IH have further corroborated these disruptions, showing varied gene expression changes including increased expression of genes such as *PER2*, *PER3*, *NR1D1*, and *NR1D2* in lung cells [23] and decreased expression of *BMAL1* [24]. Despite these insights, our investigation did not observe significant changes in circadian clock gene expression, with the exception of decreased morning BMAL1 levels, pointing towards the complexity of defining circadian disruption in OSA through gene expression profiles due to heterogeneous results.

Unsurprisingly, we found that the expression of almost none of the circadian genes correlated with any of the HIF-1 subunits. The only negative correlation found was for morning HIF-1β and PER1 protein levels in OSA. This finding contrasts with our previous study, where evening levels of PER1, CRY1, and CLOCK were positively correlated to HIF-1α [8]. These differences in study outcomes may result from the characterization of the study group, as this study included the whole spectrum of OSA (AHI ≥ 5), whereas the preliminary study focused solely on the severe form (AHI ≥ 30).

It is possible that patients with varying degrees of OSA severity may exhibit distinct circadian cycle gene expression profiles. For example, in more severe cases of OSA, where hypoxic episodes are more pronounced and frequent, one might expect an upregulation of genes involved in the hypoxia-response pathway, potentially disrupting circadian regulators such as BMAL1 or PER1. Conversely, in milder cases, these disruptions may be less evident or absent. The HIF-1α-related response to hypoxia is likely a key mediator of these processes, given its role in cellular adaptation to oxygen deprivation. Indeed, HIF-1α levels have been shown to positively correlate with AHI, the primary parameter used to assess OSA severity [13]. This correlation suggests that the severity of hypoxia, as reflected by higher AHI values, drives increased HIF-1α activity, which may subsequently influence circadian clock dynamics. However, there are no comprehensive studies that systematically investigate differences in circadian clock gene expression across the spectrum of OSA severity, highlighting an important avenue for future research.

Interestingly, we did not find any correlations between genes and their products, which tips the explanation toward post-transcriptional or post-translational mechanisms of protein upregulation. In normoxia, HIF-1α is degraded in a ubiquitin-dependent way with the help of von Hippel–Lindau tumor suppressor (VHL) and prolyl hydroxylase domain (PHD) proteins. PHD hydroxylates HIF-1α, making it possible for VHL to bind and mark it for destruction. This degradation process needs oxygen, so in hypoxia, the hydroxylation of HIF-1α stops. This leads to an increase in HIF-1α protein level and its overactivity, without overexpressing its gene [25,26]. There are also other proteins responsible for ubiquitin-dependent degradation of circadian cycle proteins. Beta-transducin repeat containing E3 ubiquitin protein ligase (β-TrCP) is responsible for PER protein degradation [27]. PER is phosphorylated earlier by casein kinase 1δ/ε, which increases its affinity for β-TrCP, leading to ubiquitination and degradation by the proteasome [28]. Similarly, F-box protein 3 (FBXL3) acts in the degradation of CRY proteins. None of the above ligases has ever been studied in OSA. However, there is evidence that β-TrCP is overexpressed under hypoxia in prostate cancer cells and affects the stabilization of HIF-1α [29]. This could mean that, in OSA patients, the ligases may be dependent on oxygen levels, possibly affecting concentrations of the circadian clock proteins. This may also explain the negative correlation between morning levels of HIF-1 and CRY1 proteins. Nonetheless, the postulation above remains speculative and requires empirical verification in subsequent investigative studies. A limitation of this study is the small size of the control group (n = 16) compared to the OSA group (n = 54), which may have influenced some of the findings, such as the lack of significant changes in circadian clock gene expression (except for decreased morning BMAL1 levels) and the absence of correlations between genes and their products. While post-transcriptional or post-translational mechanisms are proposed as potential explanations, the small control group size should also be considered a possible contributing factor. This highlights the imperative for rigorous experimental validation to corroborate the hypothesized mechanisms and their physiological implications under differential oxygen availability.

It is imperative to acknowledge several limitations inherent to our investigation. Initially, the regulation of gene expression is subject to a myriad of influences, including hormonal fluctuations, metabolic conditions, and environmental variables. Consequently, the regulatory pathways of the circadian genes examined may be governed by more complex mechanisms than those addressed within the scope of this study. Furthermore, our analysis was based on blood sample collections conducted at two time points, precluding the establishment of comprehensive diurnal profiles for the circadian clock components. Additionally, the use of peripheral blood as the source of biological material, though practical, may not fully reflect the gene expression patterns within the central circadian clock located in the suprachiasmatic nucleus. Despite this, the employment of leukocytes from peripheral blood as a model for studying human circadian mechanisms remains a common approach, including within the context of our research. The final limitation is the substantial disparity in the sizes of the study and control groups and not including severities of OSA, which could potentially influence the results. Nonetheless, these factors necessitate a cautious interpretation of our findings, highlighting the need for further studies that incorporate broader evaluative criteria and methodologies to elucidate the intricate dynamics of circadian regulation in obstructive sleep apnea.

## 4. Materials and Methods

### 4.1. Sample

Seventy participants were recruited at the Sleep and Respiratory Disorders Centre in Łódź (Poland). They underwent a diagnostic attended nocturnal polysomnography (PSG) examination and, based on the results (Apnea–Hyponea Index (AHI)), were assigned to either the OSA (AHI ≥ 5) or control group (AHI < 5) [30]. Apnea is defined as the complete cessation of airflow through the nose and mouth for at least 10 s during sleep, often accompanied by a drop in oxygen saturation [31]. Hypopnea refers to a partial reduction in airflow of at least 30%, lasting at least 10 s, and typically associated with a 3–4% oxygen desaturation or an arousal from sleep [31]. Age within 18–75 years and body-mass index (BMI) between 20 and 45 kg/m^2^ were set as inclusion criteria, while individuals with inflammatory diseases (e.g., connective tissue diseases or inflammatory bowel diseases), chronic respiratory diseases (e.g., bronchial asthma or chronic obstructive pulmonary disease), any infection within one month of blood collection, diagnosis of cancer (in medical history), diagnosed major neurological conditions, diagnosed psychiatric disorders including insomnia, or taking medications affecting sleep (e.g., benzodiazepines and melatonin) were excluded from the study. The Ethics Committee of the Medical University of Lodz approved the study (RNN/432/18/KE). All participants provided written informed consent to participate in the study. All methods were performed in accordance with the relevant guidelines and regulations.

### 4.2. Polysomnography

Upon arriving at the sleep lab, participants underwent a physical examination, including measurements of body mass, height, heart rate, and blood pressure. During the recording of nocturnal polysomnography (PSG), channels were used as follows: electroencephalography (EEG), electromyography (EMG) of chin muscles and anterior tibialis, electrooculography (EOG), oronasal airflow measurements (thermistor gauge), snoring recordings, body position tracking, respiratory movements of chest and abdomen (piezoelectric gauges), unipolar electrocardiogram (ECG), and hemoglobin oxygen saturation (SpO_2_) (Alice 6, Phillips Respironics, Murrysville, PA, USA). The sleep stages were scored based on the 30-s epoch standard, as outlined in the American Academy of Sleep Medicine (AASM) guidelines [30]. Furthermore, apnea was defined as a reduction in airflow to less than 10% of baseline for at least 10 s, while hypopnea was characterized by a minimum 30% decrease in airflow for at least 10 s, accompanied by a SpO_2_ decrease of more than 3% or an arousal, which were also scored according to AASM guidelines [30].

### 4.3. Material Collection and Assessment of Protein and mRNA Levels

Blood samples were collected from participants in the evening before and morning following the sleep study using tubes equipped with clot activator and EDTA. The first collection took place 15 min before lights out (around 9:00 PM) and the second collection occurred within 10 min of awakening (around 6:00 AM). After drawing the blood, the samples containing the clot activator were immediately centrifuged at 4 °C. The resulting serum was then collected and stored at −80 °C.

The protein levels of serum BMAL1 (EIAab, Wuhan, China), CLOCK (EIAab, Wuhan, China), PER1 (EIAab, Wuhan, China), CRY1 (FineTest, Wuhan, China), HIF-1α (Invitrogen, Carlsbad, CA, USA), and HIF-1β (EIAab, Wuhan, China) were determined using enzyme-linked immunosorbent assay (ELISA) kits. The absorbance of each sample was measured at a wavelength of 450 nm using a dedicated absorbance reader, a Nanodrop Colibri Microvolume Spectrometer (Titertek Berthold, Bad Wildbad, Germany).

RNA was extracted from peripheral blood leukocytes (PBLs) using TRIzol (Invitrogen, Waltham, MA, USA). The quality of the isolated RNA was evaluated using two metrics: RNA Integrity Number (RIN) and concentration. This was performed using a high-throughput Colibri Microvolume Spectrometer (Berthold Technologies, Bad Wildbad, Germany). The isolated RNA was reverse-transcribed into cDNA using a dedicated kit according to the manufacturer’s instructions. The reverse transcription process involved three steps, including annealing the primers at 60 °C for 60 s. The level of expression of the chosen genes, *BMAL1*, *CLOCK*, *PER1*, *CRY1*, *HIF-1α*, and *HIF-1β*, was determined using quantitative real-time polymerase chain reaction (qRT-PCR). The qRT-PCR mixture contained nuclease-free water, a master mix, cDNA, gene-specific probes targeting *BMAL1*, *CLOCK*, *PER1*, and *CRY1*, *HIF-1α*, *HIF-1β*, and a reference gene, *β-actin*. Three qRT-PCR reactions were performed for each sample and the reference gene. The cycle threshold (CT) was determined for each sample and the reference gene. The difference in CT values (∆Ct) was then calculated and used to analyze mRNA expression levels. The mRNA expression level was calculated using the following equation: 2^−∆Ct^, and presented following multiplication by 10.

### 4.4. Statistical Analysis

Data analysis was conducted using SPSS 28.0 (IBM, Armonk, NY, USA). The distribution of variables was assessed using the Shapiro–Wilk test. Parameters with normally distributed data were compared using a paired *t*-test and independent student *t*-test, while Wilcoxon and Mann–Whitney U tests were employed for parameters with non-normal distributions. Continuous data with distributions other than normal were presented as median and interquartile range (IQR), while normally distributed data were presented as mean and standard deviation. Spearman’s rank correlation was used to assess relationships between variables. The level of statistical significance was set at *p* < 0.05.

## 5. Conclusions

Our study confirms the complexity of the regulation of biological clock proteins and HIF-1α in the context of OSA, suggesting that these interactions may involve both known and as yet unidentified posttranscriptional and posttranslational mechanisms. It establishes the presence of a strong relationship between circadian clock elements and subunits of HIF-1 among OSA patients highlighting the importance of hypoxia in circadian regulation, since these associations were not observed in the control group. We found increased levels of CLOCK, CRY1, PER1, and HIF-1α proteins in OSA patients, yet our gene expression results do not fully match observations at the protein level, highlighting the need for further research to more fully understand these processes. Moreover, the lack of a significant correlation between circadian clock gene expression and protein levels of HIF-1 subunits, with isolated exceptions, indicates the complexity of the interactions between these systems. In light of our findings and the existing literature, it is clear that future research should focus on further deciphering this dynamic regulatory network, which may ultimately lead to new therapeutic strategies for treating OSA and related disorders.

## Figures and Tables

**Table 1:** Demographic and polysomnographic characteristics of the study groups. AHI, apnea–hypopnea index; BMI, body mass index; OSA, obstructive sleep apnea; nREM, non-rapid eye movement; REM, rapid eye movement; SpO_2_, oxygen saturation; TST, total sleep time. The bold text in the table indicates statistical significance.

Parameter	Control Group (n = 16)	OSA Group (n = 54)	*p*-Value
Age, years old	49.9 SD 9.2	51.9 SD 11.6	0.53
BMI, kg/m^2^	30.4 SD 6.1	32.7 SD 5.4	0.14
Sex, male [n]	81.3% (n = 13)	92.6% (n = 50)	0.34
Sleep Efficiency, %	80.8 (71.9–88.2)	86.4 (74.3–90.7)	0.24
Sleep Onset Latency, min	17.8 (10.6–25.9)	13.3 (8.0–24.0)	0.19
Sleep Maintenance Efficiency, %	86.9 (82.4–92.3)	92.1 (80.0–95.3)	0.18
REM Sleep Latency, min	78.5 (56.6–99.8)	81.0 (60.8–137.3)	0.28
TST, h	6.0 SD 1.30	6.2 SD 0.9	0.33
REM Duration, h	1.3 SD 0.60	1.2 SD 0.5	0.33
nREM Duration, h	4.6 SD 0.8	5.0 SD 0.8	0.06
Arousal Index, events/h	14.0 (6.5–23.2)	18.2 (11.4–27.9)	0.06
AHI, events/h	1.0 (0.5–1.8)	26.1 (13.9–56.3)	**<0.001**
AHI in REM, events/h	1.2 (0.0–3.9)	29.5 (11.7–46.9)	**<0.001**
AHI in nREM, events/h	1.0 (0.4–1.4)	21.7 (10.8–50.6)	**<0.001**
Total number of desaturations	10.0 (6.0–15.0)	136.0 (73.3–313.8)	**<0.001**
Desaturation Index, events/h	2.0 (1.0–2.8)	33.5 (17.0–61.4)	**<0.001**
Basal SpO_2,_ %	93.3 (92.4–94.2)	91.5 (89.7–93.1)	**<0.001**
Mean SpO_2_ during desaturations, %	90.6 (88.5–92.5)	86.8 (83.3–89.2)	**<0.001**
Minimum SpO_2,_ %	88.9 (84.5–91.4)	75.0 (65.5–81.3)	**<0.001**

**Table 2:** Comparisons of circadian clock gene and HIF-1 subunit gene expression and protein levels. Correlations between circadian clock gene expressions and protein levels in the evening and the morning. BMAL1, brain and muscle ARNT-like 1; CLOCK, circadian locomotor output cycles kaput; CRY1, cryptochrome 1; HIF-1α, hypoxia-inducible factor 1 subunit α; HIF-1β, hypoxia-inducible factor 1 subunit β; OSA, obstructive sleep apnea; PER1, period 1. The bold text in the table indicates statistical significance.

Parameter			Control Group	OSA Group	*p*-Value Control Group vs. OSA Group	*p*-Value Evening vs. Morning Control Group	*p*-Value Evening vs. Morning OSA Group
Gene expression	evening	BMAL1	6.10 (3.78–15.48)	6.38 (2.13–29.87)	0.80	0.39	0.39
	morning		14.53 (6.58–26.55)	6.31 (1.28–14.03)	**0.02**		
	evening	CLOCK	3.70 (1.29–5.71)	2.29 (1.33–4.667)	0.35	0.20	0.29
	morning		4.78 (1.76–16.13)	3.33 (1.35–6.29)	0.20		
	evening	CRY1	4.05 (2.12–7.03)	1.84 (0.82–4.80)	**0.09**	0.26	0.06
	morning		6.60 (1.97–12.40)	3.58 (1.60–5.92)	0.12		
	evening	PER1	2.05 (0.96–6.96)	2.63 (0.71–9.59)	0.91	**0.02**	**0.004**
	morning		6.90 (5.26–27.86)	7.45 (2.96–16.29)	0.49		
	evening	HIF-1α	25.12 (7.49–28.80)	19.74 (3.98–42.97)	0.66	0.69	0.94
	morning		1620 (7.99–43.22)	17.92 (5.56–36.16)	0.95		
	evening	HIF-1β	14.21 (7.96–35.50)	16.28 (7.65–45.60)	0.99	0.40	0.49
	morning		35.12 (13.53–57.20)	22.41 (7.37–42.74)	0.23		
Protein level	evening	BMAL1, ng/mL	17.41 (17.07–18.60)	17.54 (17.06–18.02)	0.86	0.21	0.92
	morning		17.62 (16.97–17.97)	17.62 (17.25–17.96)	0.59		
	evening	CLOCK, ng/mL	3.51 (3.22–3.62)	3.66 (3.53–4.02)	**0.03**	0.92	0.89
	morning		3.49 (3.21–3.59)	3.66 (3.56–4.00)	**0.008**		
	evening	CRY1, ng/mL	35.30 (21.81–43.11)	38.15 (30.93–48.65)	**0.048**	0.15	0.78
	morning		27.95 (18.44–39.83)	37.71 (32.10–43.78)	**0.003**		
	evening	PER1, ng/mL	243.05 (185.06–294.42)	304.20 (254.93–381.10)	**0.003**	0.35	0.86
	morning		246.52 (212.66–305.47)	304.09 (261.43–384.30)	**0.02**		
	evening	HIF-1α, ng/mL	2.41 (2.20–2.93)	2.73 (2.29–3.41)	0.25	0.22	0.58
	morning		2.36 (2.15–2.60)	2.78 (2.30–3.53)	**0.04**		
	evening	HIF-1β, ng/mL	70.80 (69.62–72.36)	71.90 (68.87–72.60)	0.53	0.06	0.22
	morning		70.27 (67.09–72.19)	71.94 (69.853–72.42)	0.19		

**Table 3:** Correlations between circadian clock gene expressions and protein levels in the evening and the morning. Weak correlation: |R| < 0.3, moderate correlation: 0.3 ≤ ∣R∣ < 0.5, strong correlation: ∣R∣ ≥ 0.5. BMAL1, brain and muscle ARNT-like 1; CLOCK, circadian locomotor output cycles kaput; CRY1, cryptochrome 1; HIF-1α, hypoxia-inducible factor 1 subunit α; HIF-1β, hypoxia-inducible factor 1 subunit β; OSA, obstructive sleep apnea; PER1, period 1. The bold text in the table indicates statistical significance.

			Gene Expression				Protein Level			
			HIF-1α		HIF-β		HIF-1α		HIF-β	
			Evening	Morning	Evening	Morning	Evening	Morning	Evening	Morning
OSA group	BMAL1	evening	**R = 0.457** ***p* = 0.003**		**R = 0.363** ***p* = 0.02**		R = −0.228 *p* = 0.10		R = −0.076 *p* = 0.59	
		morning		**R = 0.504** ***p* < 0.001**		**R = 0.474** ***p* < 0.001**		R = 0.035 *p* = 0.80		R = 0.170 *p* = 0.22
	CLOCK	evening	**R = 0.312** ***p* = 0.046**		**R = 0.355** ***p* = 0.03**		R = −0.195 *p* = 0.16		R = 0.155 *p* = 0.26	
		morning		**R = 0.441** ***p* = 0.002**		**R = 0.608** ***p* < 0.001**		R = −0.136 *p* = 0.33		R = 0.029 *p* = 0.84
	CRY1	evening	**R = 0.581** ***p* < 0.001**		**R = 0.616** ***p* < 0.001**		R = −0.168 *p* = 0.23		R = 0.155 *p* = 0.26	
		morning		**R = 0.521** ***p* < 0.001**		**R = 0.465** ***p* = 0.001**		R = −0.252 *p* = 0.07		R = 0.029 *p* = 0.84
	PER1	evening	**R = 0.356** ***p* = 0.02**		**R = 0.653** ***p* < 0.001**		R = 0.053 *p* = 0.71		R = −0.233 *p* = 0.09	
		morning		R = 0.050 *p* = 0.73		**R = 0.424** ***p* = 0.003**		R = −0.053 *p* = 0.71		**R = −0.322** ***p* = 0.02**
Control group	BMAL1	evening	R = 0.473 *p* = 0.09		**R = 0.692** ***p* = 0.01**		R = 0.090 *p* = 0.74		R = −0.099 *p* = 0.74	
		morning		R = 0.107 *p* = 0.70		R = 0.314 *p* = 0.25		R = 0.149 *p* = 0.58		R = −0.288 *p* = 0.32
	CLOCK	evening	**R = 0.571** ***p* = 0.03**		**R = 0.874** ***p* < 0.001**		R = 0.338 *p* = 0.20		R = −0.112 *p* = 0.70	
		morning		R = −0.020 *p* = 0.95		**R = 0.550** ***p* = 0.03**		R = −0.100 *p* = 0.71		R = −0.165 *p* = 0.57
	CRY1	evening	**R = 0.552** ***p* = 0.04**		R = 0.522 *p* = 0.07		R = −0.053 *p* = 0.85		R = −0.086 *p* = 0.77	
		morning		R = 0.279 *p* = 0.32		R = 0.564 *p* = 0.03		**R = −0.588** ***p* = 0.02**		R = 0.218 *p* = 0.46
	PER1	evening	R = 0.246 *p* = 0.38		R = 0.187 *p* = 0.54		R = 0.265 *p* = 0.32		R = −0.275 *p* = 0.34	
		morning		R = −0.175 *p* = 0.53		R = 0.471 *p* = 0.07		R = 0.094 *p* = 0.73		R = −0.209 *p* = 0.47

## Data Availability

The datasets generated and/or analyzed during the current study are available from the corresponding author upon reasonable request.
